# Histologic and Biochemical Effect of *Balanite aegyptiaca* Fruit Extract on Alloxan-Induced Diabetes in Wistar Rats

**DOI:** 10.4314/ejhs.v33i3.7

**Published:** 2023-05

**Authors:** Solomon Matthias Gamde, Chinenye J Ugwah-Oguejiofor, Aminu Garba, Godwin O Avwioro, Akinpelu Moronkeji, Abdullahi Abiodun Jimoh

**Affiliations:** 1 Department of Medical Laboratory Science, Bingham University Karu, Nasarawa State, Nigeria; 2 Department of Pharmacology & Toxicology, Usmanu Danfodiyo University, Sokoto, Nigeria; 3 National Health Insurance Scheme Authority, Sokoto State Office, Nigeria; 4 Department of Medical Laboratory Science, Delta State University Abraka, Nigeria; 5 Department of Medical Laboratory Science, University of Medical Sciences, Ondo City, Nigeria; 6 Department of Medical Laboratory Science, Usmanu Danfodiyo University, Sokoto, Nigeria

**Keywords:** Diabetes mellitus, Toxicity, Balanite aegyptiaca, Healing

## Abstract

**Background:**

Diabetes mellitus is among the most prevalent and costly chronic diseases in the world. Unfortunately, immediate prospects for a cure are not available. We aimed to determine the in vivo antidiabetic activity, histologic, and biochemical effect of Balanites aegyptiaca fruit extract on alloxan-induced diabetes in Wistar rats.

**Methods:**

Thirty-six Wistar rats were allotted into six groups (n=6). Group I was normal control. Group II was induced with diabetes but not treated.Groups III-V were induced with diabetes and treated with 100, 200, and 300 mg/kg extracts while Group VI was treated with Metformin once daily for 14 days. Animals were euthanized, and blood samples were collected for biochemical assays. The liver, kidney, pancreas, and testis were excised and processed by the paraffin wax method.

**Result:**

Oral administration of BA extract significantly (P<0.05) reduced blood glucose, liver enzymes, and creatinine levels in diabetic animals. The extract also improved the body weights of diabetic animals and microscopic anatomy of the pancreas, testis, liver, and kidney parenchyma compared to the control.

**Conclusion:**

Balanites aegyptiaca phytochemicals reduced blood glucose level and improved the histology of the liver, kidney, pancreas, and testis. Further study is recommended to identify the phytochemicals and mechanism of action.

## Introduction

Diabetes mellitus is among the most prevalent and costly chronic diseases in the world (1). It's characterized by high blood glucose levels due to insulin secretion deficiency, insulin action impairment, or even both (2,3). In 2021, the International Diabetes Federation (IDF) estimated that 537 million people have diabetes compared to 422 million in 2014 and 451 million in 2017. By 2030, this number is expected to rise to 643 million and 783 million by 2045 (4). This figure is a gross underestimate because the number of people living with diabetes is largely unknown to the World Health Organization's African Region (5).

Despite continuous efforts made by health systems to reduce the prevalence of diabetes that is thought to be preventable has been unsatisfactory. Different studies have assessed different models that predict the complications of patients with diabetes (6,7,8). Unfortunately, immediate prospects for a cure are not available. Drug resistance is a major problem opposing synthetic drugs, and due to this phenomenon, people will continue to be affected by diabetes and its growing prevalence in the aging population (9). This problem has stimulated researchers all over the world to search for alternative treatments for diabetes that are thought to be preventable.

To obtain a distinct solution, an ethnopharmacological survey represented the first and most important reference point in unveiling the treasure of natural resources (1,10). Some of these medicinal plants include *Anacardium occidentalle* (11), *Garcinia kola* (12), *Balanite agyptiaca* (13), and *Parinari curetellofolia* (14). However, the use of *Balanites aegyptiaca* (BA) for treatments has continued without sufficient information.

*Balanites aegyptiaca* (Family Balantiaceae) is traditionally used in Africa, South Asia, and the Middle East for diabetes (15,16). The fruit mesocarp is used as a raw drug for hyperglycemia and hyperlipidemia (17,18). Previous scientific studies have documented the activities of BA against ulcers (19), intestinal worms (20), malaria (21), and the *in vitro* antidiabetic activity verified (22), but there is a need to determine its antidiabetic activity *in vivo*. This study aimed to determine the *in vivo* antidiabetic activity, histologic, and biochemical effect of *Balanites aegyptiaca* fruit extract on alloxan-induced diabetes in Wistar rats.

## Materials and Methods

**Plant material**: *B. aegyptiaca* fruits were collected from Medicinal Plant Garden at the Department of Pharmacognosy, Usmanu Danfodiyo University, Nigeria. The plant was identified by Dr. H.E Mshelia, Department of Pharmacognosy and Ethnomedicine, and deposited at the herbarium with registration number UDUD HREC 2021. The fruit pericarp was washed and prepared into coarse powder as described by Hanan *et al.* (18). Seven hundred grams of the powdered fruit was macerated in 1000 ml distilled water for 24 hours on a mechanical shaker and filtered by using Whatman no. 1 filter paper and exposed to evaporation using hot plate at 40°C to obtain 47.1 g unadulterated fruit extract.

**Ethical statement**: The study was conducted following internationally-accepted principles for laboratory animal use and care. The license number of the experimental animals, UDUD HREC 09/09/2022, was provided by the Research Ethics Committee of the Usmanu Danfodiyo University. All animal operations complied with the guidelines National Institutes of Health (NIH) guidelines for the care and use of laboratory animals.

**Chemicals used**: Alloxan monohydrate (Chemical Co. St. Louis, MO, USA) and analytical grade reagents kits (Randox Laboratories Limited, Crumlin, and County Antrim, United Kingdom) for AST, ALT, AP, and E/U/Cr were purchased from Ali Shuaibu diagnostics, Sokoto in Nigeria.

**Animals used**: Thirty-six white Wistar rats weighing 160g±20 were purchased from the Animal House, Faculty of Pharmaceutical Sciences, Usmanu Danfodiyo University, Sokoto, Nigeria. The animals were housed in cages and maintained under the standard husbandry condition (between 22°C, 12 hours light and 12 hours dark) in the Animal House. Animals were fed with chow and water *ad libitum*. The use of experimental animals followed the Animal Ethical Committee of the Pharmacology Department in University of Jos approved animal studies.

**Induction of diabetes**: Five percent solution of the Alloxan monohydrate was prepared and used to induce diabetes type 2 via intraperitoneal injection at a dose of 150 mg/kg body weight of animals which fasted for 16 hours. The blood glucose level was measured before the commencement of experiment and 72 hours after alloxan administration using a glucometer. Animals whose blood glucose levels were greater than 200 mg/dL were included in test groups as described with little modification (23).

**Animals grouping and dosing**: Thirty-six animals were randomly allotted into six groups consisting of six animals each. Group I (normal control) was not induced with diabetes; Group II (Alloxan-induced diabetic control) was induced with diabetes but not treated; Groups II-V were induced with diabetes and treated daily with 100, 200, and 300 mg/kg extract, while Group VI was treated with metformin respectively for two weeks.

**Sample collection and preparation**: After the last dose, the animal's body weights were recorded using a weighing balance (CS 200, China). The animals were euthanized and blood samples were individually collected via cardiac puncture into plain bottles and allowed to clot, centrifuged, and the resultant sera were harvested for biochemical assay. The liver, kidney, pancreas, and testis were excised via abdominal incision and processed using the paraffin wax method (24).

**Assessment of serum biochemical indexes**: Liver transaminases aspartate aminotransferase (AST), alkaline phosphatase (ALP), alanine aminotransferase (ALT), total protein (TP), albumin (Alb), direct bilirubin (DB), total bilirubin (TB), urea, creatinine (Cr) and electrolytes, potassium (K^+^), sodium (Na^+^), chloride (Cl^-^), and bicarbonate (HCO_3_) were assessed using diagnostic kits from Randox laboratory, United Kingdom.

**Histopathological assessment**: The liver, kidney, pancreas, and testis were fixed in 10% buffered formalin for 24 hours. After dehydration by three changes of ethanol, clearing in xylene, and embedding in molten paraffin, 3 µm of the paraffin mass was cut into a section using a microtome (Surgcare Microtome, Model 335A USA). Cut sections were deparaffined and stained with hematoxylin and eosin (H&E) for the observation of histopathological changes (25).

**Statistical analysis:** All statistical data were analyzed using IBM SPSS (version 25) by one-way analysis of variance (ANOVA) and Bonferroni post hoc test. All tests were considered significant at *P*<0.05 compared to the control.

## Results

**Effect of BA extract anthropometric indexes**: Table 1 shows the effect of BA fruit extract on anthropometric indexes of alloxan-induced diabetes. Significant (*P*<0.05) changes were recorded in the body weights and basal metabolic index (BMI) of diabetic animals compared to normal control. After treatment with the extract, animals recorded a dose-dependent increase in BMI as compared to the control. The mean body weights of diabetic animals treated with extract and metformin were significantly (*P*<0.05) lower than the normal control (Table 1).

**Table 1:** Effect of BA extract on Anthropometric indexes

Group	Weight (g)	Height (cm)	BMI (g/cm^2^)
**Normal control**	143.75 ± 21.08	11.25 ± 1.73	12.80 ± 2.98#
**DM only**	96.50 ± 6.50*	7.15 ± 0.35	18.90 ± 2.22*
**DM+100 mg/kg BA**	92.67 ± 4.49*	6.71 ± 0.14*	20.60 ± 2.31*#
**DM+200 mg/kg BA**	104.20 ± 5.25*#	7.05 0.22*	21.00 ± 2.98*#
**DM+300 mg/kg BA**	131.00 ± 7.00*#	6.60 ± 0.50*	30.35 ± 2.43*#
**DM+50 mg/kg Met.**	78.00 ± 3.39*#	6.25 ± 0.21*	20.25 ± 2.96*#

Data were expressed as mean value ± standard error of the mean (SEM).

* Mean values were significantly different compared to the Normal control at *P*≤ 0.05.

# mean values were significantly different compared to the diabetes group (Alloxan-induced DM only) at *P*≤0.05.

DM means diabetes Mellitus and BA means *Balanite aegyptiaca*.

**Figure 2:**
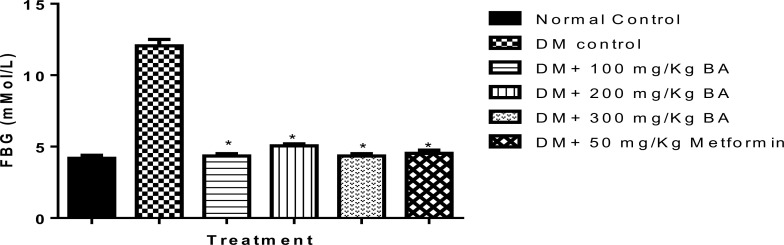
The effect of BA extract on blood glucose levels; *mean values were significantly different compared to the diabetes group at P≤ 0.05; DM means diabetes mellitus; BA means Balanite aegyptiaca; FBG means fasting blood glucose

**Effect of BA extract on blood glucose**: Administration of alloxan significantly raised the blood glucose levels compared to the normal group (*P*<0.05), indicating a significant increase in fasting blood glucose (Figure 2). However, oral administration of 100, 200, and 300 mg/kg extract significantly (*P*<0.05) reduced the fasting blood glucose (FBG) levels. Compared with the standard drug metformin, the blood glucose levels were also significantly (*P*<0.05) reduced. There was no significant difference in the glucose levels of treated animals to control. The extract better-reduced blood glucose levels than metformin.

**Effect of BA extract on liver function markers**: Table 2 shows the effect of BA on liver function markers. Alloxan induced a significant (*P*≤0.05) rise in serum transaminases (ALP and ALT) and bilirubin levels. In contrast, BA extract significantly (*P*<0.05) reduced the raised serum transaminases (ALP and ALT) and bilirubin levels compared to the diabetic group. There was a dose-related increase in serum albumin and total protein after extract administration of 100, 200, and 300 mg/kg to diabetic animals.

**Table 2:** Effect of BA extract on liver function markers

Group	AST (U/L)	ALP (U/L)	ALT (U/L)	DB(mg/dL)	TB (mg/dL)	TP (g/L)	ALB (g/L)
Normal control	4.75 ± 0.85	5.00 ± 1.08	72.00±6.92	0.18 ± 0.05	0.43 ± 0.06	67.50±1.94	41.00±3.63
DM only	10.50±1.00*	10.50±1.50*	86.50±8.50*	0.50± 0.01*	0.90± 0.10*	54.01±1.00*	36.02±6.30*
DM+100mg/kgBA	8.83 ± 1.11*	7.30±1.02*#	78.17±6.31*#	0.28±0.04*#	0.67±0.11*#	65.33±3.43*#	34.00±2.71*
DM+200 mg/kg BA	8.60 ± 0.93*	8.41 ± 1.05*#	64.31±6.42*#	0.26±0.25*#	0.66±0.05*#	68.08±4.11*#	35.04± 1.30*
DM+300 mg/kg BA	9.50 ± 2.50*	8.01 ± 3.02	73.20±4.00#	0.20±0.01*#	0.75± 0.15	74.00±7.02#	37.50 ± 2.50
DM+50 mg/kg Met.	8.50 ± 0.87*#	7.00 ± 1.58*#	81.25±9.39*#	0.28±0.05*#	0.83± 0.15*	71.31±1.96*#	37.25 ±1.75*

Data were expressed as mean value ± SEM.

* Mean values were significantly different compared to the normal control group at *P*≤0.05.

# Mean values were significantly different compared to the diabetes group (Alloxan-induced DM only) at *P* ≤ 0.05.

**Effect of BA on kidney function markers**: Table 3 shows the effect of BA extract on kidney function markers. Serum levels of creatinine were significantly (*P*<0.05) elevated in diabetic animals (DM only). However, administrations of BA caused a significant (*P*<0.05) dose-related decrease in the creatinine levels as compared to the diabetic control. Changes in serum urea, sodium, potassium, and bicarbonate ions were non-statistically significant (*P*>0.05).

**Table 3:** Effect of BA extract on kidney function markers

Group	Na^+^ (mMol/L)	K^+^ (mMol/L)	Cl (mMol/L)	HCO_3_ (mMol/L)	Urea (mMol/L)	Cr (mg/dL)
Normal control	139.25 ± 1.38	4.15 ± 0.26	100.25± .14	27.51 ± 1.85	4.30 ± 0.34	0.48 ± 0.11
DM only	141.51 ± 7.50	3.45 ± 0.25	93.50 ± 6.50	32.00 ± 1.02	6.05 ± 0.15	1.00±0.11*
DM+100mg/kg	137.83 ± 2.75	3.50 ± 0.17	95.00 ± 2.73	26.31 ± 1.24	5.05 ± 0.54	0.83 ± 0.11
DM+200mg/kg BA	135.60 ± 4.09	3.04 ± 0.37	90.70 ± 1.25	25.60 ± 1.61	4.60 ± 0.43	0.62 ± 0.66
DM+300mg/kg BA	141.50 ± 3.50	4.50 ± 0.40	104.50±0.50	28.00 ± 3.12	4.41 ± 0.73	0.50 ±0.21#
DM+50mg/kg Met	142.53 ± 3.12	3.73 ± 0.37	97.75 ± 2.25	26.25 ± 2.25	5.28 ± 0.33	0.78 ± 0.09

Data were expressed as mean value ± SEM.

* Mean values were significantly different compared to the normal control group at *P*≤0.05.

# Mean values were significantly different compared to the diabetes group (Alloxan-induced DM only) at *P*≤0.05.

**Figure 3a-f:**
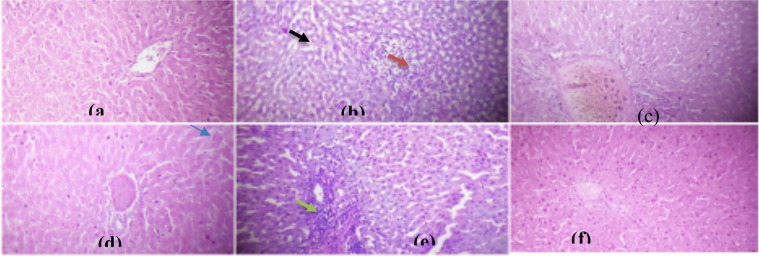
(a) normal liver cells, diabetic control liver (b) indicated inflammation (red arrow) and apoptotic cells (black arrow); (c-e) diabetic animals treated with 100, 200, and 300 mg/kg BA rejuvenated the liver cells as compared to normal and metformin (f) g; 300 mg/kg BA(e) incites severe inflammatory cells (green arrow) (H&E. X Mag. 400.)

**Figure 4a-f:**
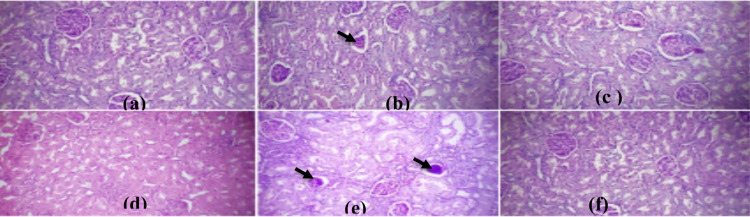
(a) normal kidney parenchyma; (b) diabetic kidney exhibited glomerular shrinkage (black arrow); (c-e) diabetic animals treated with 100, 200, and 300 mg/kg BA repaired the glomerular shrinkage similar to metformin (f); (e) 300 mg/kg BA caused glomerular shrinkage (black arrow) (H&E. X Mag. 400)

**Figure 5a-f:**
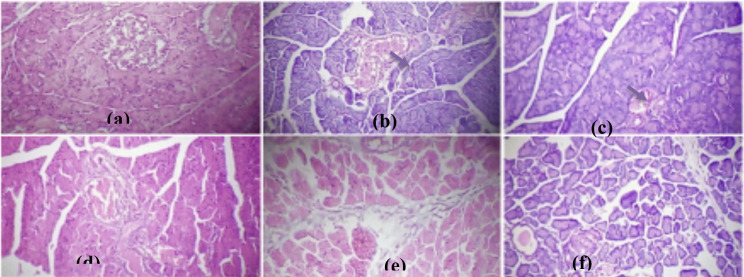
(a) normal pancreatic cell, (b) diabetic pancreas illustrated diminished islet cells (blue arrow), (c-e) diabetic animals treated with 100, 200, and 300 mg/kg indicated recuperating islets cells restoring functional β-cells; the activity was higher than the standard drug metformin (f) (H&E. X Mag. 400).

**Figure 6a-f:**
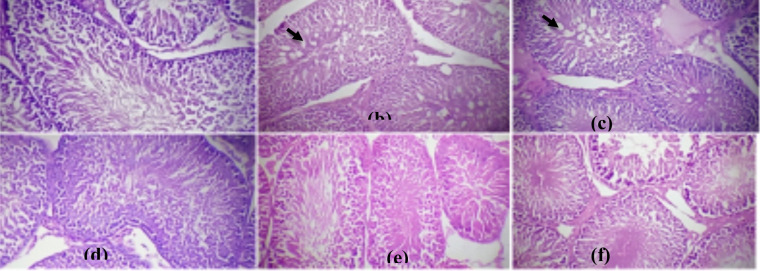
(a) normal testicular cell, (b) diabetic animal demonstrated disorganized and vacuolated seminiferous tubules (black arrow); (c-e) diabetic animals treated with 100, 200, and 300 mg/kg BA revealed a dose-related testicular repair similar to metformin (f) (H&E. X Mag. 400.

**Histopathological assessment of the organs**: Figures 3-6 show the histopathology of the liver, kidney, pancreas, and testis of normal, diabetes, and animals treated groups. Histopathological assessment of diabetic animals showed apoptotic liver cells with inflamed cells. BA administered at 100 and 200 mg/kg improved the injured liver cells. However, a high dose of 300 mg/kg extract triggered inflammatory cells.

Diabetic animals exhibited glomerular shrinkage, but BA administered at 100 and 200 mg/kg overhauled the glomerular shrinkage similar to the control. In contrast, 300 mg/kg extract caused glomerular shrinkage.

Diabetic animals showed diminished islet cells. BA administered at 100 and 200 mg/kg improved the endocrine tissue forming islets of Langerhans thereby restoring functional β-cells. Diabetic animals further demonstrated degenerated seminiferous tubules with vacuolations in the tubules. BA administered at 100, 200, and 300 mg/kg informed a dose-related testicular repair.

## Discussion

Previous studies have documented the different health problems of uncontrolled diabetes in several biological systems (7,26,27,28). A new therapeutic modality is anticipated to prevent and eradicate the damaging effects of diabetes. In this study, alloxan administration significantly increased blood glucose levels and caused deleterious changes to the pancreas, liver, kidney, and testis. The mean body weights of diabetic animals were lower than the normal control. A decrease in relative body weight is a sign of organ toxicity associated with the degradation of tissue proteins, enhanced gluconeogenesis from muscle proteins (29), and lipolysis of triglycerides due to diabetes (30). This result is closely consistent with previous studies which showed a significant decrease in the mean body weight of diabetic animals (6,31) but contrary to Hassan *et al.* (16) which reported a significant increase in the body weight of diabetic rats. However, the administration of BA extract increased the body weight of diabetic groups in a dose-related manner including their proteinemia levels compared to the control group.

In our study, the histopathological assessment showed proof of the degenerative changes in the islets cells of the diabetes group, signifying diminished functional β-cells that are responsible for the raised blood glucose. This is consistence with previous studies that diabetes is produced by a defect of insulin action, secretion, or even both factors (32,33). However, the necrosed pancreas showed a notable regeneration after administration of BA extract at different concentrations. Our result also showed proof of the inflammatory cells and vacuolations in the liver parenchyma in tandem with the raised liver enzymes (ALT, AST, and ALP), total, and conjugated bilirubin levels in diabetic animals. The histopathological and biochemical changes that occurred in the liver are in agreement with previous studies (18,31) where there were inflammatory cells, raised serum transaminases (AST and ALT), and total and conjugated bilirubin levels in diabetic animals. The raised liver enzymes in diabetic control were due to the histopathological changes in the liver cells. Liver enzymes are highly centered in the liver and are released in the serum in significant quantities when the cell membrane becomes leaky and even ruptured (34).

Similarly, the effect of BA extract on kidney histology and function was tested. The kidney is susceptible to toxicants since a high volume of blood flows through it and its ability to filter large amounts of toxins could concentrate in the tubules (35). In this study, there was a significant increase in the level of creatinine between diabetes groups and normal control which suggests that the kidney was injured. Creatinine is a more dependable marker than urea to assess kidney function, a considerable increase in both creatinine and urea indicated that the diabetic animals suffered nephrotoxic changes. This is in agreement with the present histopathological result that illustrated diabetes-induced glomerular distortion. In addition, the present study also indicated that diabetic animals suffered testicular damage. One of the most noticeable changes occurring in the epithelial of the seminiferous tubules was extensive vacuolation in diabetic rats. This process may be due to premature exfoliation of the spermatogenic cells in the adluminal compartment leading to the development of large spaces (vacuolations) in the seminiferous epithelial (36). This is consistent with the previous finding connecting different testicular complications with diabetes syndrome inhibiting spermatogenesis (7).

In the present study, oral administration of BA indicated a promising outcome against alloxan-induced diabetes as well as the biochemical and histopathological changes in the liver, pancreas, kidney, and testis. However, it is important to note that high dose BA produced some levels of organ toxicities, and the underlying mechanisms behind the dose-time effect are yet to be understood. To identify the best resampling approach for each animal model, a depth study is needed to understand the mechanisms that drive the critical effects.

## Figures and Tables

**Figure 1:**
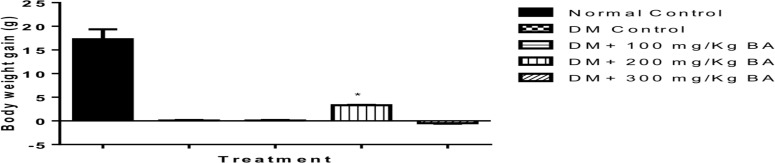
The body weight changes following BA administration.
